# Exploring the CK2 Paradox: Restless, Dangerous, Dispensable

**DOI:** 10.3390/ph10010011

**Published:** 2017-01-20

**Authors:** Cinzia Franchin, Christian Borgo, Silvia Zaramella, Luca Cesaro, Giorgio Arrigoni, Mauro Salvi, Lorenzo A. Pinna

**Affiliations:** 1 Department of Biomedical Sciences, University of Padova, via U. Bassi, 58/B, 35131 Padova, Italy; cinzia.franchin@unipd.it (C.F.); christian.borgo@unipd.it (C.B.); luca.cesaro.1@unipd.it (L.C.); giorgio.arrigoni@unipd.it (G.A.); mauro.salvi@unipd.it (M.S.); 2 Proteomics Center, University of Padova and Azienda Ospedaliera di Padova, via G. Orus, 2/B, 35129 Padova, Italy; silviazaramella7@gmail.com; 3 CNR Neurosciences Institute, via U. Bassi, 58/B, 35131 Padova, Italy

**Keywords:** protein kinase CK2, casein kinase 2, cancer, signal transduction, non oncogene addiction, phosphoproteomics, CRISPR/Cas9 technology

## Abstract

The history of protein kinase CK2 is crowded with paradoxes and unanticipated findings. Named after a protein (casein) that is not among its physiological substrates, CK2 remained in search of its targets for more than two decades after its discovery in 1954, but it later came to be one of the most pleiotropic protein kinases. Being active in the absence of phosphorylation and/or specific stimuli, it looks unsuitable to participate in signaling cascades, but its “lateral” implication in a variety of signaling pathways is now soundly documented. At variance with many “onco-kinases”, CK2 is constitutively active, and no oncogenic CK2 mutant is known; still high CK2 activity correlates to neoplasia. Its pleiotropy and essential role may cast doubts on the actual “druggability” of CK2; however, a CK2 inhibitor is now in Phase II clinical trials for the treatment of cancer, and cell clones viable in the absence of CK2 are providing information about the mechanism by which cancer becomes addicted to high CK2 levels. A phosphoproteomics analysis of these CK2 null cells suggests that CK2 pleiotropy may be less pronounced than expected and supports the idea that the phosphoproteome generated by this kinase is flexible and not rigidly pre-determined.

## 1. Background

The discovery of the first phosphoprotein dates back to 1883 when Olof Hammarsten demonstrated that the milk protein casein contains almost 1% of tightly bound phosphorous [1]. In retrospect, this finding gave rise to one of the most teasing “cold cases” of biochemistry, considering that the enzyme(s) responsible for casein phosphorylation remained unknown for 130 years. In fact, none of the hundreds of protein kinases detected in the second half of the past century and forming the so-called “kinome” is responsible for the physiological phosphorylation of casein. The “genuine” casein kinase (“G-CK”) remained an orphan enzyme until 2012 when it was identified with Fam20C, an atypical protein kinase responsible for the generation of a large proportion of the phosphosecretome [2,3,4].

In the meantime, however, casein had been successfully used as an artificial substrate for the characterization of many other protein kinases, with special reference to those denoted by the acronyms CK1 (with several isoforms) and CK2, reminiscent of the misnomers “casein kinase” -1 and -2. These two were responsible for the first “protein phospho kinase” activity ever described, isolated in 1954 from rat liver [5] and later shown to be ubiquitously present in many other organisms and tissues. CK2, in particular, is the subject of this special issue.

The rising interest for this unique kinase, as predicted by the Nobel laureate Edwin Krebs in a 1999 paper entitled “CK2, a protein kinase of the next millennium” [6], is largely accounted for by two features of this enzyme: outstanding pleiotropy and pathogenic potential. The former implies that an increasing number of researchers are “coming across” CK2 in the course of their investigations, the latter justifying the numerous efforts to dissect signaling pathways perturbed by abnormal CK2 activity and to develop therapeutic strategies aimed at its downregulation. Both aspects are amply documented by the multi-author contributions [7] and are dealt with in this special issue. Here, we present evidence that our current view about CK2 pleiotropy and indispensability may need to be reconsidered.

## 2. Pleiotropy

**Figure 1:**
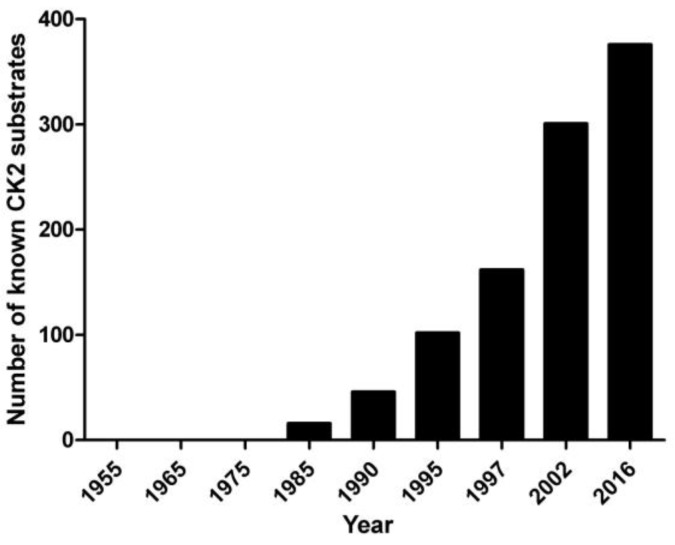
The growing number of proteins phosphorylated by CK2 since its discovery in 1954. Constructed with data from [10,11,12,13]. According to PhosphoSitePlus [10], the number of phosphosites known to be generated by CK2 is presently 640, which belong to almost 400 proteins altogether.

The paradox of CK2 pleiotropy is illustrated in Figure 1, illustrating that CK2 remained a kinase in search of its substrates for two decades after its discovery; the first were detected in the 1980s, followed by a snowball effect. Today, there are almost 600 phosphosites generated by CK2 according to PhosphoSitePlus, making it the second most pleiotropic member of the kinome (see also Table 1). This may be just the tip of the iceberg if we trust bioinformatics approaches based on the WebLogo of the whole phosphoproteome, where the features of typical CK2 sites are so remarkable that its contribution can reach 20% of all phosphosites [8,9].

This figure is probably an overestimate, as we will see later, but we must consider that CK2 pleiotropy does not mean only many substrates, but also a plethora of interactors as reviewed in [14], and implication in many signaling pathways [15].

In this respect, it should be borne in mind that the mode of implication of CK2 in these pathways is unusual: not being activated by either phosphorylation or specific stimuli, its hierarchical participation in cascades, like other kinases, is hardly conceivable; in fact, its role is that of a lateral player, impinging “horizontally” on a variety of “vertical” signaling cascades, as discussed elsewhere [16,17].

## 3. Pathogenic Potential

Coincidental evidence of the oncogenic potential of CK2 was provided in the 1980s, as documented in a 1993 review quoting 11 reports where CK2 was invariably higher in a variety of tumors as compared to normal tissues/cells [18]. This repertoire was further implemented at later times [19,20]. In the meantime, work in David Seldin’s lab demonstrated a cause–effect link between CK2 upregulation and transformation induced by oncogenes or by deficiency of tumor suppressor genes [21,22,23]. More recently, an analysis in the oncomine database revealed that the main CK2 catalytic subunit α is overexpressed in 5 out of the 6 most important types of cancer in the US [24].

The oncogenic potential of CK2 is challenged by the observation that, unlike other onco-kinases generated by gain of function mutation conferring constitutive/unscheduled activity, CK2 is constitutively active by itself, and no gain of function CK2 mutations are known to be responsible for neoplastic transformation. Thus, the rising concept is that elevated CK2 level makes the cellular environment more favorable to malignant transformation by the mechanism known as “non-oncogene addiction” [16]. Indeed, many of the effects of abnormally high CK2 are expected to potentiate the cancer phenotype, with special reference to its strong pro-survival and anti-apoptotic efficacy [25].

Addiction can therefore be simplistically depicted as a process by which cells stochastically enriched in CK2 are selected by the tumor itself, because they escape apoptosis offering a kind of “sanctuary” to malignancy. The term “oncophilic” has been coined to denote these cells particularly susceptible to malignant transformation [9]. CK2 blockage can therefore revert or even eradicate the cancer phenotype relying on abnormally high CK2.

Consistently, in many cases, cancer cells have been shown to be more sensitive to the cytotoxic efficacy of CK2 inhibitors than their normal counterparts, thus giving rise to a long and continuously increasing list of cells that are known to critically rely on CK2 for their survival.

CK2 is not only implicated in neoplasia but also in several other human diseases [15]. Of note in particular, recent reports have highlighted the role of CK2 in ischemia [26,27], thrombosis [28,29,30], diabetes [31], and inflammation induced by TNF-alpha [32].

## 4. Druggability

The pathogenic potential of CK2 accounts for many efforts done during the past two decades to develop cell permeable inhibitors of this kinase. Hundreds of these compounds have been described in the literature, mostly, but not exclusively, competitive with respect to ATP. A recent repertoire of these compounds is available in [33], and some of these are dealt with in this special issue. The amount of work done in this field is further documented by the more than 70 structures of CK2 in complex with its inhibitors deposited in PDB and by the observation that some of these have been already tested for their tolerability in animal models.

Despite considerable efforts to develop CK2 inhibitors, pharmaceutical companies have been reluctant to invest on CK2 druggability due to a number of properties of this kinase. One was the lack of any clear-cut connection between CK2 and individual pathways whose dysregulation specifically promotes malignancy. The other two were the striking pleiotropy of CK2, suggesting that its inhibition is likely to cause too many collateral effects, and its purported essential role, documented by lethality during embryogenesis of animals where its subunits had been knocked out [34,35]. The first caveat has been slowly overcome by the rising concept that, although CK2 may be not a *sensu stricto* oncogene, causative of malignancy by itself, many tumors are addicted to its abnormally high activity, rendering its downregulation a valuable multi-purpose anticancer strategy. On the other hand, it is conceivable that, although reliance on CK2 is critical during embryogenesis, CK2 reduction might be tolerated in adult cells. Consistent with this suspicion, a breakthrough was made in 2010 by the CK2 inhibitor CX-4945 (also known as Silmitasertib) that entered clinical trials for the treatment of different kinds of cancer [36] and is now in Phase II [37].

## 5. Dispensability

More recently, the proof of concept that CK2 may be dispensable for life has been provided by the generation of viable cell clones where both its catalytic subunits were knocked out by CRISPR(clustered regularly interspaced short palindromic repeats)/Cas9 technology [38,39]. A quantitative MS analysis indicates that these CK2 null cells cope with the lack of CK2 by undergoing proteomics alterations that reflect a functional rewiring expectedly adverse to malignant transformation.

Interestingly, the proteomics alteration induced by a pharmacological inhibition of CK2 is only partially overlapping the perturbation observed in CK2 null cells [39], thus corroborating the view that transient suppression of CK2 catalytic activity does not entirely account for the functional and metabolic rewiring underwent by cells deprived of this pleiotropic kinase. In other words, it is clear from the data available that cellular adaptation to CK2 suppression implies deep and complex re-adjustments, where failure to phosphorylate individual targets represents only one side of the coin.

From a practical standpoint, CK2 null cells will provide a valuable tool to address the crucial issue of off-target effects displayed by CK2 inhibitors. None of these in fact is endowed with absolute specificity, and even the first-in-class of these compounds, CX-4945, already in clinical trials, has been shown to affect splicing in a CK2-independent manner [40]. Therefore, viable CK2 null cells represent an excellent model where to dissect biological functions whose pharmacological alteration is either mediated or not by CK2.

**Figure 2:**
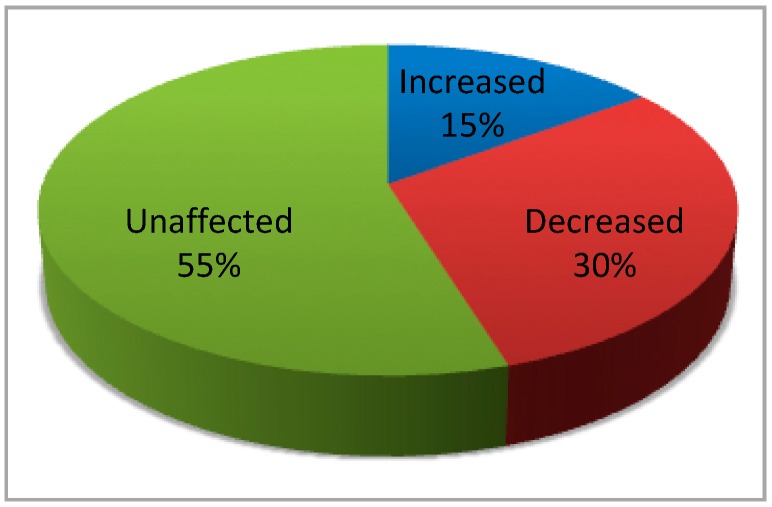
Only a minor proportion of the phosphosites, conforming to the CK2 consensus quantified in C2C12 myoblasts devoid of both CK2 catalytic subunits, is drastically reduced (>50%), as compared to the wild type. A SILAC experiment has been conducted on C2C12 cell lines where both the catalytic CK2 **α** subunits have been knocked out by the CRISPR/Cas9 technology, as described in Borgo et al. [39]. CK2 null cells have been compared with wild type, both from the proteomics [39] and the phosphoproteomics side (unpublished data). To determine which phosphosites are affected by the absence of CK2, phosphopeptide enrichment has been performed, as already described in [41], and only peptides with alterations in the phosphorylation state of at least 50% have been considered as significantly varied.

The outcome of a parallel phosphoproteomics analysis reveals that, although a large proportion of quantified phosphosites conform to the consensus sequence of CK2 (s/t-X-X-E/D/s), only less than one third of these are significantly decreased in the CK2 null cells, while the majority are unaffected and a few are even increased (Figure 2).

This apparent paradox is accounted for by a couple of considerations. Firstly, the CK2 consensus is present in more than 3000 phosphosites (out of almost 15,000) generated by kinases other than CK2, according to the PhosphoSitePlus database [13]. In fact, as shown in Table 1, where only the most pleiotropic kinases are considered, the CK2 consensus is invariably present in some of the phosphosites generated by all of these, with a frequency sometimes higher than 20%. Secondly, the proteomics analysis of CK2 null cells [39] has led to the quantification of 26 protein kinases: although these make up just about 5% of the whole kinome, they are sufficient to account for the indirect effects of CK2 knock out mediated by other kinases. Six kinases in fact are over-expressed in CK2 null cells. Among these, Aurora B is especially noteworthy as it is over-expressed 27-fold in CK2 null cells, while a considerable number of its phosphosites also conform to the CK2 consensus (see Table 1), thus providing an example of how the absence of CK2 can promote the paradoxical effect of increasing rather than decreasing the occupancy of some phosphosites displaying the CK2 consensus.

**Figure 3:**
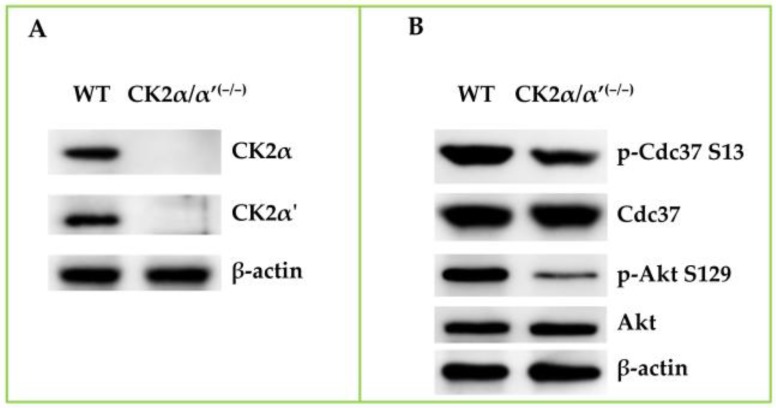
The phosphorylation of bona fide CK2 sites is not entirely abrogated in CK2 null cells. Wild-type (WT) and CK2 null (CK2α/α’^(**−**/**−**)^) cells were lysed in a buffer containing 20 mM Tris-HCl (pH 7.5), 1% Triton X-100, 10% glycerol, 1 mM EDTA, 150 mM NaCl, and protease and phosphatase inhibitor cocktails. Thirty micrograms of lysate proteins were subjected to 11% SDS-PAGE and analyzed by Western blot with the indicated antibodies. Panel **A** shows that both the CK2 catalytic subunits are absent in the CK2 null cells. In panel **B,** the significant residual phosphorylation of two bona fide CK2 sites in these cells can be appreciated. The figure is representative of three independent experiments.

On the other hand, it is remarkable that even two well established bona fide CK2 phosphosites, pS13 of CDC37 and pS129 of AKT, often used as reliable reporters of endogenous CK2 activity, do not disappear, as one would expect, in CK2 null cells, the former being only modestly reduced (Figure 3). This means that other kinases can partially replace CK2 to ensure the phosphorylation of crucial residues that are normally targeted by this kinase.

## 6. Conclusions

The data presented support two unanticipated conclusions. Firstly, the pleiotropy of CK2 appears to be less pronounced than expected since many phosphosites conforming to the CK2 consensus are in reality generated by kinases different from CK2 as their occupancy is unaffected in CK2 null cells. Secondly, it seems likely that CK2 can be replaced by other kinases to perform the phosphorylation of critical sites whenever CK2 activity is abrogated. In other words, the rising concept is that the phosphoproteome generated by CK2 is not as ample as suspected before—and more importantly, it is not rigidly pre-determined.

## Figures and Tables

**Table 1:** Presence of the CK2 consensus S/T-x-x-E/D/pS/pT at the phosphosites generated by the most pleiotropic protein kinases. Calculated from the PhosphoSitePlus database [13]. Only the 24 most pleiotropic kinases have been considered. “%” expresses the percentage of phosphosites generated by each individual kinase where the CK2 consensus is present.

Kinase	p-Sites	p-Sites with CK2 Consensus	%
PKACA	834	201	24.10
CK2A1	640	483	75.46
PKCA	637	115	18.05
CDK2	588	94	15.98
CDK1	569	86	15.11
ERK2	497	78	15.69
ERK1	375	65	17.33
Akt1	325	73	22.46
GSK3B	322	89	27.63
ATM	232	64	27.58
PLK1	229	70	30.56
P38A	226	45	19.91
CAMK2A	216	50	23.14
Chk1	205	74	36.09
CDK5	190	20	10.52
JNK1	187	43	22.99
PKCD	156	24	15.38
AurB	153	35	22.87
AMPKA1	145	36	24.82
PKCB	145	27	18.62
CK1A	139	60	43.16
DNAPK	117	38	32.47
PKCE	104	20	19.23
mTOR	99	26	26.26
